# Reconstructing the last common ancestor of all eukaryotes

**DOI:** 10.1371/journal.pbio.3002917

**Published:** 2024-11-25

**Authors:** Thomas A. Richards, Laura Eme, John M. Archibald, Guy Leonard, Susana M. Coelho, Alex de Mendoza, Christophe Dessimoz, Pavel Dolezal, Lillian K. Fritz-Laylin, Toni Gabaldón, Vladimír Hampl, Geert J. P. L. Kops, Michelle M. Leger, Purificacion Lopez-Garcia, James O. McInerney, David Moreira, Sergio A. Muñoz-Gómez, Daniel J. Richter, Iñaki Ruiz-Trillo, Alyson E. Santoro, Arnau Sebé-Pedrós, Berend Snel, Courtney W. Stairs, Eelco C. Tromer, Jolien J. E. van Hooff, Bill Wickstead, Tom A. Williams, Andrew J. Roger, Joel B. Dacks, Jeremy G. Wideman

**Affiliations:** 1 Department of Biology, University of Oxford, Oxford, United Kingdom; 2 Ecologie Systématique Evolution, CNRS, Université Paris-Saclay, AgroParisTech, Gif-sur-Yvette, France; 3 Department of Cell & Molecular Biology, The University of Rhode Island, Kingston, Rhode Island, United States of America; 4 Department of Biochemistry and Molecular Biology and the Institute for Comparative Genomics, Dalhousie University, Halifax, Canada; 5 Department of Algal Development and Evolution, Max Planck Institute for Biology Tübingen, Tübingen, Germany; 6 School of Biological and Behavioural Sciences, Queen Mary University of London, London, United States of America; 7 Department of Computational Biology, University of Lausanne, Lausanne, Switzerland; 8 Swiss Institute of Bioinformatics, Lausanne, Switzerland; 9 Charles University, Faculty of Science, Department of Parasitology, BIOCEV, Vestec, Czech Republic; 10 Department of Biology, University of Massachusetts Amherst, Amherst, Massachusetts, United States of America; 11 Barcelona Supercomputing Centre (BSC-CNS), Barcelona, Spain; 12 Institute for Research in Biomedicine (IRB Barcelona), The Barcelona Institute of Science and Technology, Barcelona, Spain; 13 Catalan Institution for Research and Advanced Studies (ICREA), Barcelona, Spain; 14 CIBER de Enfermedades Infecciosas, Instituto de Salud Carlos III, Madrid, Spain; 15 Hubrecht Institute-KNAW, Oncode Institute, UMC Utrecht, Utrecht, the Netherlands; 16 Institut de Biologia Evolutiva (CSIC-Universitat Pompeu Fabra), Barcelona, Spain; 17 Okinawa Institute of Science and Technology Graduate University (OIST), Okinawa, Japan; 18 Department of Evolution, Ecology and Behaviour, Institute of Infection, Veterinary and Ecological Sciences, University of Liverpool, Liverpool, United Kingdom; 19 Department of Biological Sciences, Purdue University, West Lafayette, Indiana, United States of America; 20 Department of Ecology, Evolution and Marine Biology, University of California, Santa Barbara, California, United States of America; 21 Centre for Genomic Regulation (CRG), Barcelona Institute of Science and Technology (BIST), Barcelona, Spain; 22 Theoretical Biology and Bioinformatics, Department of Biology, Faculty of Science, Utrecht University, Utrecht, the Netherlands; 23 Department of Biology, Lund University, Lund, Sweden; 24 Cell Biochemistry, Groningen Biomolecular Sciences and Biotechnology Institute, Rijksuniversiteit Groningen, Groningen, the Netherlands; 25 Laboratory of Microbiology, Wageningen University & Research, Wageningen, the Netherlands; 26 School of Life Sciences, University of Nottingham, Nottingham, United Kingdom; 27 School of Biological Sciences, University of Bristol, Bristol, United Kingdom; 28 Division of Infectious Diseases, Department of Medicine, and Department of Biological Sciences, University of Alberta, Edmonton, Canada; 29 Institute of Parasitology, Biology Centre, Czech Academy of Sciences, České Budějovice, Czech Republic; 30 Centre for Life’s Origins and Evolution, Department of Genetics, Evolution, & Environment, University College, London, United Kingdom; 31 Center for Mechanisms of Evolution, School of Life Sciences, Arizona State University, Tempe, Arizona, United States of America

## Abstract

Understanding the origin of eukaryotic cells is one of the most difficult problems in all of biology. A key challenge relevant to the question of eukaryogenesis is reconstructing the gene repertoire of the last eukaryotic common ancestor (LECA). As data sets grow, sketching an accurate genomics-informed picture of early eukaryotic cellular complexity requires provision of analytical resources and a commitment to data sharing. Here, we summarise progress towards understanding the biology of LECA and outline a community approach to inferring its wider gene repertoire. Once assembled, a robust LECA gene set will be a useful tool for evaluating alternative hypotheses about the origin of eukaryotes and understanding the evolution of traits in all descendant lineages, with relevance in diverse fields such as cell biology, microbial ecology, biotechnology, agriculture, and medicine. In this Consensus View, we put forth the status quo and an agreed path forward to reconstruct LECA’s gene content.

## Abstract 2

Understanding the origin of eukaryotic cells is one of the most difficult problems in all of biology. This Consensus View lays out guidelines to identify the gene repertoire of the Last Eukaryotic Common Ancestor (LECA), which will be crucial to evaluate alternative hypotheses about the origin of eukaryotes and the evolution of traits in all descendant lineages, something relevant to many fields across the life sciences.

## Introduction

The origin of the eukaryotic cell is one of the most significant evolutionary transitions in the history of life [1]. Eukaryotes are fundamentally different from their prokaryotic relatives (Bacteria and Archaea) in how the cell is organised, how these cells “feed,” move, and respond to stimuli, and how their genes are structured and expressed. Eukaryogenesis is a subject of active research and debate [2–9]). Because the eukaryotic cell evolved between 1.5 and 2.5 billion years ago [10–12], direct experimental approaches are limited and phylogenetic analyses are vulnerable to methodological artefacts [13–15]. These are problems compounded by having no other major transition of a similar age and complexity to which eukaryogenesis can be compared. Consensus on how eukaryotes first arose is thus lacking, and it is unclear how best to approach unanswered questions in order to maximise the effectiveness of future research.

Debates about eukaryogenesis span multiple disciplines including microbiology, paleobiology, and cell biology; yet they often rely heavily on phylogenomic investigations [16]. These analyses involve inferring the distribution and evolutionary history of gene families across eukaryotic and prokaryotic diversity. Here, we provide recommendations for establishing a robust phylogenomics-based picture of the genetic, metabolic, and cellular repertoires of the ancestral form(s) that gave rise to all extant eukaryotes, i.e., the last eukaryotic common ancestor (LECA) [17,18]. The goal is to produce a resolved picture of LECA and a tractable gene repertoire. The latter will serve as an important data set for understanding the prokaryotic origin-(s) of the eukaryotes and to compare different hypotheses pertinent to early eukaryotic cell evolution.

### A minimal consensus on the origin of eukaryotes

Most researchers accept that LECA originated after an association of at least 2 organisms descending from prokaryotes of evolutionarily distinct lineages—one arising from within the Archaea [19,20], likely within the Asgardarchaeota [8,21], and the other related to Alphaproteobacteria [21–24]. We refer to this scenario as the “two+” model of eukaryogenesis (i.e., 2 partners coupled with significant evolutionary change in the fundamental cell biology of this emerging form). This baseline scenario provides a starting point for comparing alternative hypotheses. For example, many variant hypotheses suggest that additional lineages contributed to eukaryogenesis [25], e.g., a “third partner” arising from deltaproteobacteria [26] or chlamydia-like bacteria [27], while others have suggested an alternative starting point to eukaryogenesis from close to the planctomycetes [28]. Still others have suggested that viruses were major contributors [29–33], although viruses of various forms certainly acted as agents moving genes between lineages throughout the history of eukaryotic evolution [30,34], thus making it difficult to identify early viral contributions to eukaryogenesis. Which auxiliary lineages participated, when and how—either by bursts of horizontal gene transfer (HGT) from short-lived microbial associations, or longer-term integrations like the endosymbiotic processes that led to the mitochondrion or the plastid [22,35]—are long-standing questions in the eukaryogenesis debate.

Although there is broad acceptance that living eukaryotes arose from a common ancestor that had genetic and cellular features of mixed archaeal and bacterial ancestry, hypotheses differ as to which cellular lineage is proposed to have “encapsulated” the other; some suggest an archaeon took up a bacterium [36–40] while others argue the opposite [26]. Other models envisage the alphaproteobacterium-related mitochondrion as having been established via phagocytosis by a proto-eukaryote of archaeal ancestry that already possessed many of the canonical features of extant eukaryotes, such as a cytoskeleton, endomembrane system, and nucleus [41]. These various models have been discussed extensively (e.g., [21,26,36–40,42,43]) with little resolution.

Several contributions have sought a clear definition of terms relating to eukaryogenesis to help frame wider debate [21,44–46]. The First Eukaryotic Common Ancestor (FECA) can be defined as the first descendant—on the eukaryotic side—of the last common ancestor of an Asgardarchaeota lineage and the eukaryotes [44,46] (i.e., the first organism whose living descendants only include eukaryotes and no other extant lines). Under the two+ model at least one other FECA lineage can be said to have existed, i.e., the first descendant of the last common ancestor of the alphaproteobacteria-related progenitor and the eukaryotes [23,24,44,46]. To simplify discussion, we refer to this latter FECA as the first mitochondrial common ancestor or FMCA (pronounced “Firmca”) [21]. There could be additional FECAs if a third or even fourth lineage were also involved in eukaryogenesis as suggested by some analyses [47]. The divergences of eukaryotes from Asgard archaea and from Alphaproteobacteria are important because they mark the beginning of the period in which the hallmark features of eukaryotes might have evolved. However, crucially and perhaps counterintuitively, there is no implication that archaeal FECA or FMCA were more eukaryote-like than their immediate prokaryotic ancestors, because the cellular features we now associate with eukaryotes might have evolved at any point on the stems between either the archaeal FECA and LECA or between FMCA and LECA [46].

At present, the unresolved gap between the archaeal FECA and LECA, and indeed FMCA and LECA, makes it difficult to infer the order and nature of events between these ancestral forms [21]. Additional sampling of lineages that branch closer to the eukaryotes than currently known prokaryotes would add greater resolution in understanding eukaryogenesis. Attempts have been made to reconstruct the order of prokaryotic gene acquisition (e.g., Asgard, alphaproteobacterial, or additional prokaryotic contributions) between these 2 points [47,48], but our understanding of this process remains limited. Analyses of shared gene content between Asgardarchaeota and extant eukaryotes have been useful in gaining a clearer picture of one set of contributions to LECA [8,9,49]. However, reconstructing the contribution of any FECA—including FMCA—depends on knowing the gene content of LECA.

### How can reconstruction of LECA inform our understanding of eukaryogenesis?

In order to appropriately understand LECA, 2 related problems need to be addressed:

- What was the molecular cell biology of LECA? Specifically, what molecular components and cellular systems evolved prior to LECA? Which of those systems arose later, as the eukaryotic lineages diverged?
- Where did LECA come from? Specifically, which prokaryotic subgroups were the key partners and which genes did they contribute? Conversely, which genes evolved de novo during the FECA-to-LECA transition/s?

If we can achieve consensus on these points, understanding LECA would enable us to define the endpoint of eukaryogenesis. This would be the end state at which all eukaryogenesis models must arrive and a starting point for understanding the evolution of the major eukaryotic groups and the cellular systems that arose within them (i.e., a baseline comparator for polarising all subsequent evolutionary transitions).

**Fig 1:**
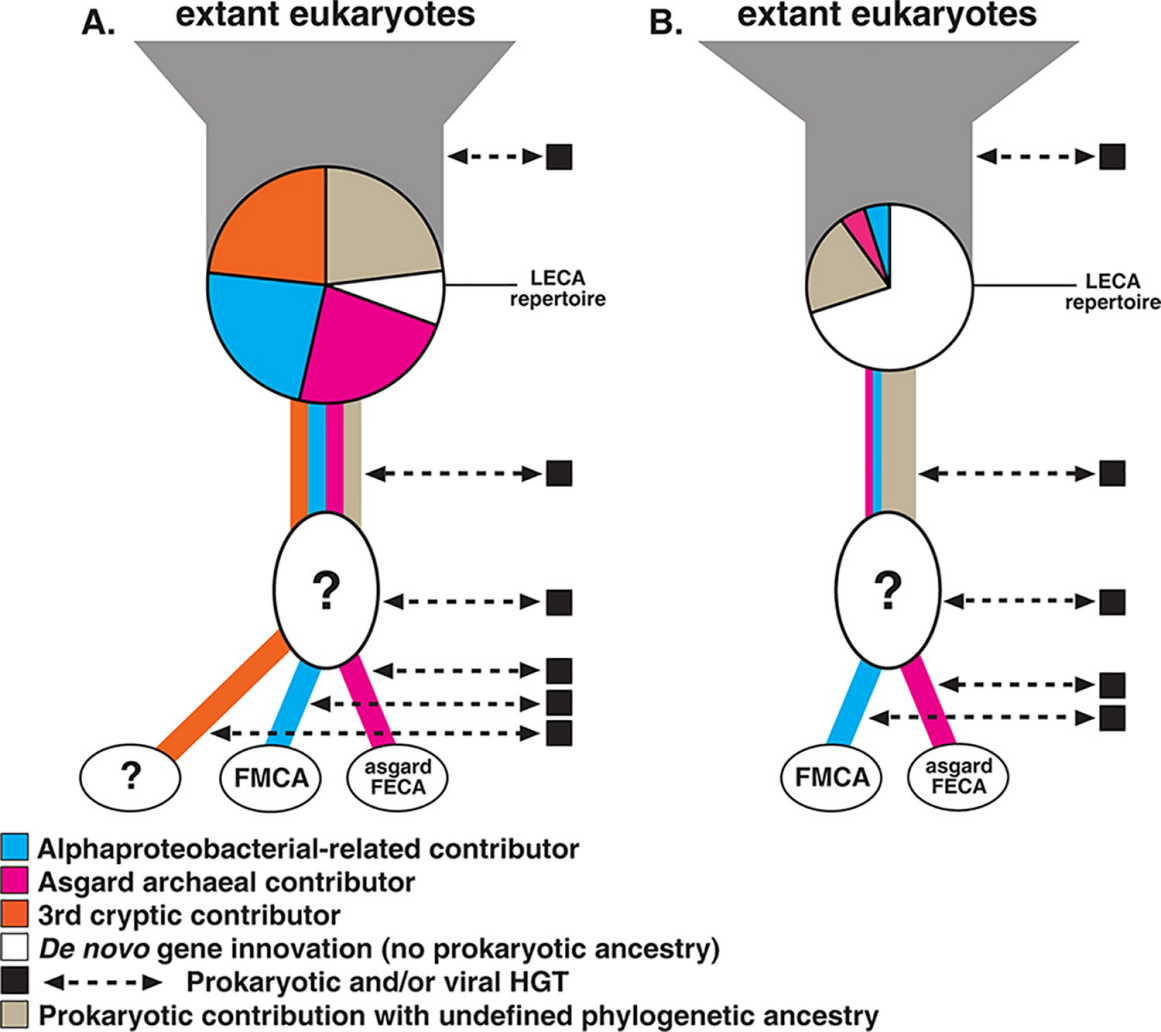
Genetic contributions to LECA. LECA’s gene repertoire was chimeric, containing genes derived from the Asgardarchaeota-derived host cell, mitochondrial endosymbiont, and potentially other prokaryotic sources, along with a set of eukaryote-specific genes that evolved after the divergence of eukaryotes from prokaryotes. The number of sources, and the proportions and identities of genes from each source, remain uncertain but can be investigated using the approach articulated in the main text of this paper. Here, we illustrate 2 possible LECA reconstructions that are broadly compatible with what is currently known about eukaryotic gene origins. **(A)** Shows a larger LECA gene repertoire reconstruction as indicated by the large pie chart. Such an inference may be the result of relatively few gene innovations post LECA, as indicated by the modest expansion after LECA leading to extant eukaryotic diversity. This hypothetical model also shows strong Asgardarchaeota and alphaproteobacterial signals and a strong additional signal from a “third party” contributor. This “third signal” could be used to argue for the role of 3 contributing lineages to eukaryogenesis beyond the two+ model. Here, the fraction of genes of de novo gene evolution (i.e., bona fide ESPs) is relatively small. The proportion of gene families of prokaryotic ancestry with poor phylogenetic resolution is not a dominant ancestral signal. **(B)** Shows a smaller LECA gene repertoire reconstruction as indicated by a smaller pie chart. Such an inference may indicate a larger-scale gene innovation post LECA, as indicated by the wider expansion after the LECA lineage leading to extant eukaryotic diversity. In this hypothetical model, the LECA repertoire with identifiable prokaryotic origin is dominated by genes of undefined ancestry. This model also shows that the LECA gene families of de novo gene ancestry (ESPs) is extensive. Only a tiny proportion of gene families present in LECA can be accurately attributed to either the Asgardarchaeota or the Alphaproteobacteria. The question marks inside the ovals on both models **A** and **B** indicate an unknown order of contribution and/or unknown contributing lineages. Dashed double arrow-headed lines indicate possible HGT contributions throughout eukaryogenesis and subsequent diversification of eukaryotes. Not all aspects of these models are mutually exclusive; for example, a large LECA repertoire (as shown in A) could be combined with a two+ model for ancestry (as shown in B). ESP, eukaryote signature protein; HGT, horizontal gene transfer; LECA, last eukaryotic common ancestor.

A consensus LECA gene repertoire also provides a framework for judging the relative merits of different eukaryogenesis models. Specifically, we can use these data to determine if “eukaryogenesis model X” has merit (utility) because it is consistent with the inferred evolutionary histories of the genes present in the LECA gene repertoire. For example, if a pattern of “third-party” ancestry (e.g., deltaproteobacteria or chlamydia [26,27,43,50,51]) is identified in a significant proportion of LECA gene trees (e.g., **Fig 1A**), then a three-partner eukaryogenesis model could then be favoured. We note that without evidence of an endosymbiotically derived compartment or genome, it would not be possible to distinguish between bursts of gene transfer from transient microbial associations, or a longer-term integration similar to the process which generated the endosymbiotically derived organelles. However, such patterns may theoretically be distinguishable from serial HGT processes as identified, for example, from viral contribution (e.g., [34]) using phylostratigraphy-like approaches [52]. However, if a substantial third-party prokaryotic signal is absent (e.g., **Fig 1B**), phylogenetic patterns provide little support beyond the two+ model.

Fig 1 compares a range of possible outcomes from LECA analyses, not just the presence or absence of a third-party contributor. For example, a relatively large LECA gene repertoire (Fig 1A) versus a smaller one (Fig 1B), implies a very different relative role for gene family gain and expansion post LECA. Furthermore, the models demonstrate very different roles for de novo gene evolution and the relative contribution of prokaryotic genes. For simplicity, these factors are shown in 2 distinct constellations. This is not to say that these are the only constellations possible—indeed different combinations of the characteristics illustrated across the 2 models can be imagined. This is not to trivialise the problem or the complexity of the data; there is a range of possible outcomes, and LECA reconstructions may identify a result somewhere between the 2 extremes shown in **Fig 1**. Our goal is to outline how different models might be supported, refuted, and appropriately modified in response to data, thereby minimising polarised debates about what genes, molecular systems, and cellular processes were—and were not—“important” for eukaryogenesis. A community-wide effort to define LECA will permit informed comparisons of different models so that they can be judged on their relative merits.

**Box 1: What do we know about LECA?**

**Fig 2:**
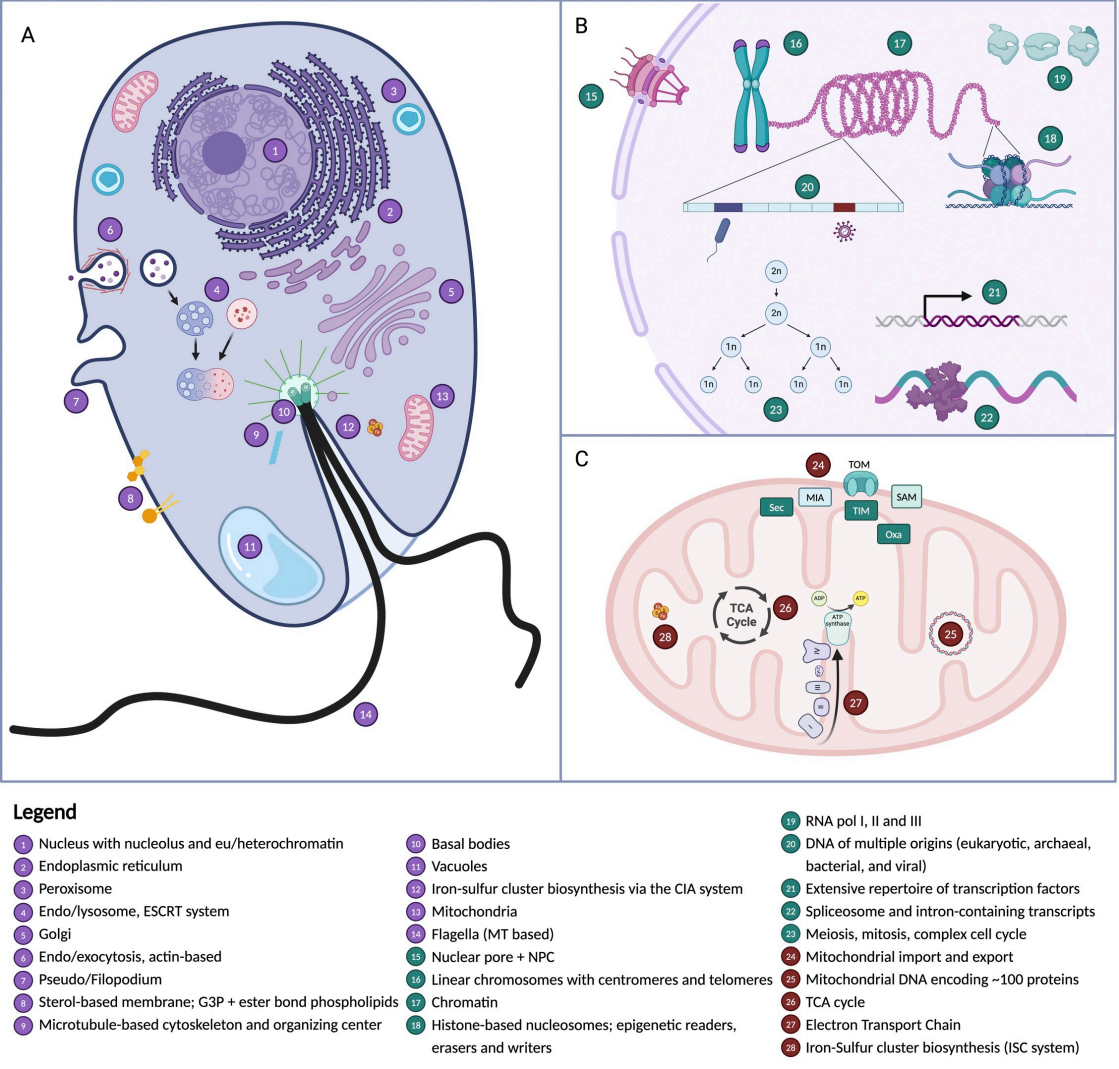
Cellular features inferred to be present in LECA. This schematic follows on from [17] and summarises the cellular features discussed in the section titled “What do we know about LECA?” (and references therein). Note that the process of meiosis, mitosis, cell division, associated machines, and processes, inferred to have been present in LECA, are not shown here. Created in BioRender. Eme, L. (2024) https://BioRender.com/w64x492. LECA, last eukaryotic common ancestor.

LECA reconstruction studies have largely focused on either cellular system-by-system analyses or investigations that take stock of total gene repertoire (e.g., [48,53]). System-specific analyses have demonstrated that LECA possessed: (i) a nucleus, nucleolus, nuclear lamina, and nuclear pore complexes [54–57]; (ii) a complex actin- and tubulin-based cytoskeleton including associated motor proteins and the systems to encode flagella, pseudo/filopodia [58–62], and mitosis encompassing a complex cell replication cycle [63–66]; (iii) genes necessary for meiosis and a facultative sexual cycle [53,67–70]; and (iv) a complex and diversified endomembrane and endomembrane trafficking system [71–74]. LECA is also inferred to have had: (v) histone/nucleosome-based chromatin with H2A, H2B, H3, and H4 paralogs and chromatin-associated catalytic functions such as methyltransferases, modification readers, and erasers [75,76], as well as SMC-based higher-level chromatin organization [77,78]; (vi) a largely archaeal-derived DNA replication system diversified by gene duplications [79,80] with some eukaryotic-specific additions (but see [32]); (vii) a spliceosome and a diversified repertoire of introns [81–86]; (viii) linear nuclear chromosomes with centromeres and telomeres [87,88] and with multi-layered regulation of gene expression [89–91]; (ix) membranes composed of fatty acid chains linked to a glycerol-3-phosphate (G3P) head group via ester bonds [92] and containing diverse sterols [93]; (x) peroxisomes [94]; and (xi) a fully integrated mitochondrial organelle similar to those found in extant lineages, with its own genome [95–100]. The population of cells that approximately constituted LECA thus had a fully fledged and elaborate eukaryotic molecular and cellular biology (**Fig 2**), not unlike many extant heterotrophic flagellated protists [17,18,101]. These patterns do not mean, however, that these core systems are immutable. Indeed, replacements, modifications, and reductions of these systems have occurred frequently across the eukaryotic tree. These include, for example, losses of flagella [102,103], peroxisomes [104], and phagocytosis [105,106], loss or radical modification of mitochondria [107–111], and the depletion of histones [112,113]. A robust LECA gene set is essential if we are to understand and appropriately account for secondary loss in eukaryotic evolution.

### Understanding the mixed ancestry of LECA

Of the fraction of genes present in LECA that possess obvious prokaryotic homology, only a small fraction can be definitively shown to be of alphaproteobacterial or asgardarchaeal origin. In a recent study of gene family evolution in eukaryotes [48], 10,233 Pfam domain families were inferred to be present in LECA. Of these, 4,335 families were acquired from prokaryotic sources, and 77% of these acquisitions were identified as having bacterial ancestry; 7% appeared to be of alphaproteobacterial-like origin. Approximately 16% of the prokaryotic acquisitions were identified as “archaeal” with only 7% specifically of Asgardarchaeota ancestry [48]. However, raw percentages do not necessarily linearly correlate with evolutionary importance. Few gene acquisitions can give rise to fundamental systems; consequently, comparisons using such statistics have to be considered carefully. Nonetheless, such data have profound implications for the two+ basic model and suggest a LECA model more closely aligned with Fig 1B (a scenario in which the ancestry of most prokaryotic genes cannot be traced back to specific donors, e.g., the Asgardarchaeota or the Alphaproteobacteria) rather than Fig 1A. What might this mean for eukaryogenesis?

The large number of LECA genes that do not trace back to either Asgardarchaeota or Alphaproteobacteria has been interpreted as evidence for additional or alternative prokaryotic or viral contributors to LECA (e.g., [25–31]). However, the presence of additional genomes and/or compartments within the eukaryotic cell, separate from the nucleus, and hosting these genes (like mitochondria and plastids), would provide indisputable evidence for additional prokaryotic partners. In the absence of such evidence, an alternative explanation is that early eukaryotic forms engaged in HGT [114] both into and out of the FECA-to-LECA lineages, a pattern seen in extant eukaryotes [115–119]. An additional variant of the HGT explanation is that these detected prokaryotic ancestries are footprints of prior transient endosymbiotic associations that laid the groundwork for the eventual mitochondrial endosymbiosis, as seen in more recent symbiotic associations and organelle acquisition events [120,121]. Another, not mutually exclusive, explanation is the “fluid prokaryotic chromosome model,” which posits that HGT between prokaryotes has been so frequent and ongoing that the genomes of the 2 prokaryotic lineages constituting the two+ model were themselves highly mosaic at the time of eukaryogenesis. More generally, incomplete taxon sampling and/or complex patterns of gene retention and loss since eukaryogenesis likely contributed to the mixed prokaryotic phylogenetic affinities seen in extant eukaryotes [122,123].

Many genes inferred to have been present in LECA do not currently have identifiable prokaryotic homologs (e.g., [48,63–65,124]). Such genes encode possible “eukaryotic signature proteins” or ESPs [39,124–127]. For example, in a 2021 study by Vosseberg and colleagues [48], 58% of the eukaryotic gene families analysed had no identifiable prokaryotic ancestry, a number that is likely to be further revised as methods change and more prokaryotes (and eukaryotes) are sampled. This reinforces the view that eukaryogenesis was a radical transition that triggered—and indeed was to a certain extent enabled by—gene family expansion. However, the discovery that Asgardarchaeota possess a subset of the genes previously classified as ESPs has somewhat altered this picture [8,9,21,49]. Nonetheless, numerous proteins not yet found in the Asgardarchaeota remain as candidate ESPs. So where did the significant proportion of LECA genes with no apparent similarity to prokaryotic genes come from? Beyond de novo gene evolution (i.e., new genes arising from non-coding DNA), it is possible that an unsampled (or extinct) third-party “prokaryotic” donor group possesses (or possessed) genes uniquely shared with the eukaryotes. It is also likely that a high rate of sequence evolution at eukaryogenesis currently prevents us from identifying the prokaryotic homologs of many ESPs based on sequence similarity alone.

A final consideration when trying to understand the ancestry of the genetic constituents of LECA is the limitations of current phylogenetic methods. Even the best methods currently available may struggle to model sequence evolution accurately over the timescales needed to understand LECA [128–131]. Phylogenomic analysis is vulnerable to artefacts [13–15] and understanding the proportion of gene families for which the signal is saturated and therefore prone to artefacts will be important to consider when evaluating support for different eukaryogenesis models (**Fig 1**). As a consequence, obtaining sufficient phylogenetic resolution for many gene families adds a considerable margin of error to any estimates for the ancestry of the LECA gene repertoire. Indeed, one of the most important results stemming from any study of LECA and eukaryogenesis would be to determine what proportion of the LECA gene set is reliable for phylogenetic inference beyond the eukaryotic clade and thus potentially useful for distinguishing between alternative hypotheses of gene ancestry.

Despite more than 2 decades of research, no data sets define the gene family repertoire that would help us to reconstruct the widest characteristics of LECA and evaluate eukaryogenesis hypotheses. This limits our ability to quantitatively estimate contributions to the stem lineages between FECA(s) and LECA from different sources, through either HGT or additional endosymbiotic partners. The absence of these data also prevents us from understanding the roles of evolutionary phenomena such as de novo gene evolution, gene fusion, and gene duplication. Understanding such phenomena requires resolved data sets and detailed approaches (e.g., [132]). We therefore argue that it is not possible to rigorously address the origin of eukaryotes without a quantitative assessment of the gene repertoire of LECA.

### Gene duplication—A further complexity in understanding LECA

**Fig 3:**
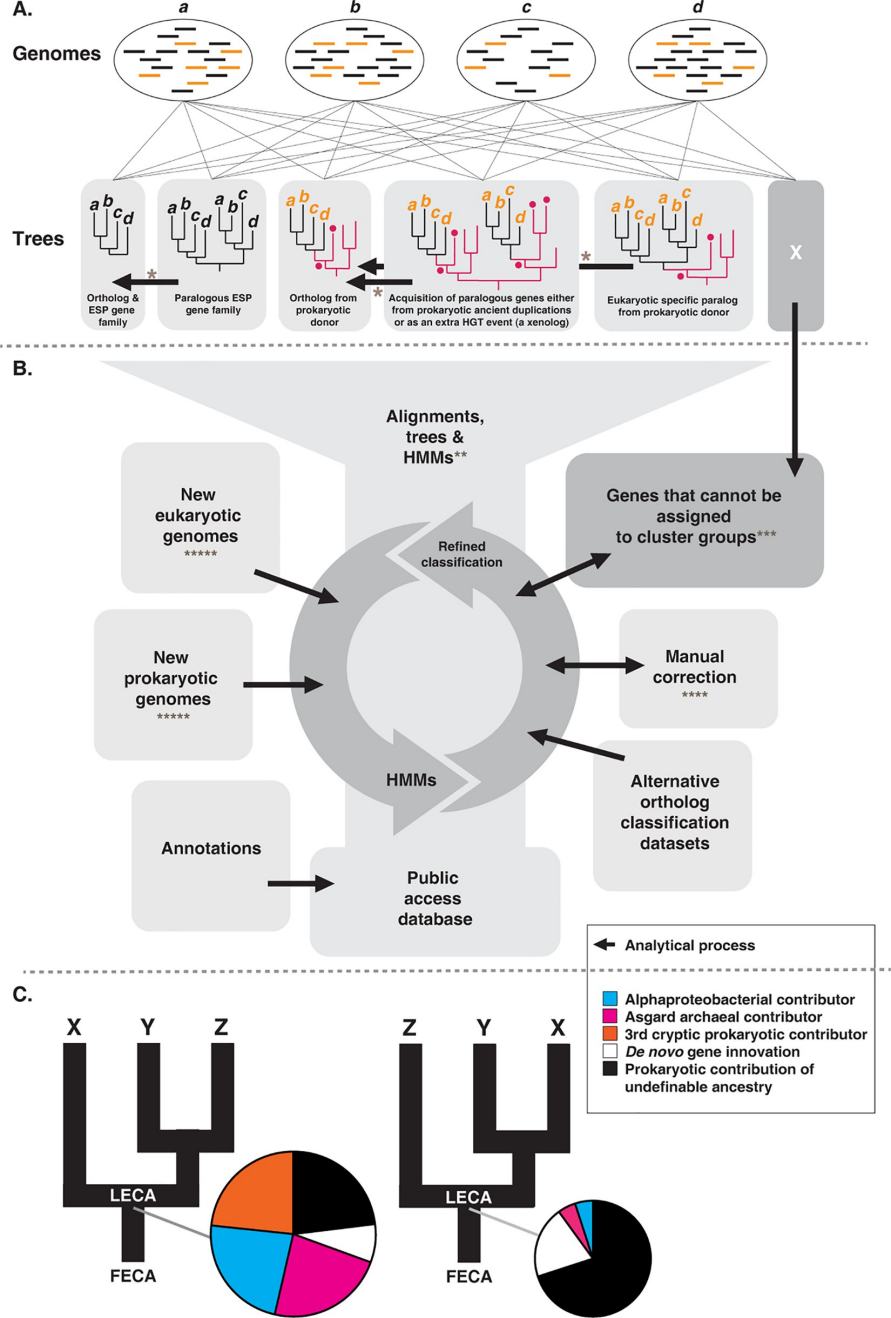
Proposed LECA gene repertoire analysis pipeline. **(A)** Eukaryotic gene complements are divided into candidate ortholog groups using phylogenetic trees. Black arrows indicate how phylogenetic analyses can be used to move from gene family phylogenies to distinct ortholog groups. Black blocks indicate genes that are specific to eukaryotes (i.e., ESPs). Orange blocks indicate eukaryotic genes of prokaryotic ancestry (phylogenetic donor-relationship is identified by red branches in the trees; red discs on the tree indicate information for inferring provenance of prokaryotic ancestry, e.g., taxonomy and node support statistics). Note that numerous genes are likely to be classified as “genes that cannot be assigned to cluster groups” (marked as box X). This pool is a repository which would allow for further revision, addition of unclassified genes to new cluster groups as they arise, or subsequent inclusion within established cluster groups as more genome data are included and the HMMs are revised. The broader process would allow cross referencing of specific orthologs to larger gene clusters, thereby allowing the ultimate ancestry of ortholog families to be inferred. **(B)** Overview of analytical process that would allow community-based revision of ortholog cluster-groupings necessary for LECA gene repertoire estimations. This process is based on HMM generation and several levels of revision allowing cluster groupings to be updated with input from numerous additional sources of data (as shown). **(C)** LECA gene repertoire estimation based on ancestral state estimation and allowing for alternative eukaryotic species tree topologies. Sources of analytical challenge and error are marked using “*” convention. *Resolving gene clusters and ortholog groups will be a highly challenging due to lack of phylogenetic resolution and hidden paralogy, likely leading to a high proportion of genes that cannot be resolved to cluster or ortholog groups. It is for this reason we advocate for iterative chains of analysis allowing for appropriate identification of such gene sets and where possible revisions. **HMMs generated for ortholog groups will likely cross-sample paralogs and/or xenologs. New tools are needed to allow ortholog sampling that excludes paralogs (e.g., [174]). ***Pipelines to cluster orphan genes will be subject to high error with false clustering of unrelated genes. ****Manual correction will involve subjective error; this is unavoidable but community access to these processes is critical to allow for downstream improvement. *****The flow of new genomic data, with different assembly and annotation standards and varying sources of contamination, will be a difficult challenge to integrate while also maintaining standards for comparative analyses. Legend is shown in a box. ESP, eukaryote signature protein; HMM, hidden Markov model; LECA, last eukaryotic common ancestor.

A consideration for LECA reconstruction analyses is the accurate identification and determination of the relative contributions of gene duplication and loss [133] (i.e., paralogous gene family expansions and differential paralog loss—see **Fig 3A**). Eukaryotes have a much greater abundance of duplicate genes and functionally differentiated paralogs than do prokaryotes, demonstrating the profound significance of this process in eukaryotic evolution, before, during, and after the divergence of the major lineages in the eukaryotic tree [48]. Indeed, paralogous expansions underpin many of the LECA cellular systems discussed in **Box 1 and Fig 2**. For example, the diversification of motor proteins through gene duplication and domain recombination has been a factor in the evolution of eukaryotic cellular complexity (e.g., [59–61,134]). Furthermore, large-scale expansions have occurred in many gene families such as small GTPases [135,136], kinases [137], and transcription factors [89,90] that control eukaryotic cellular pathways. Further back in time, gene families derived from archaea (e.g., those that play roles in DNA storage and replication and protein folding) have been subject to numerous rounds of gene duplication before LECA [76,79,138]. A full understanding of the biology of LECA thus requires an accurate delineation of the role of gene duplication before and after eukaryogenesis for both the prokaryote-derived and eukaryote-specific gene families.

### How to resolve LECA: A call for cooperative action, accessible data, and a path towards reconciliation of distinct data sets

A key problem in the field of eukaryotic evolution is that the inventory of genes from across the diversity of life is incomplete and requires continual updates as new lineages are discovered, more genomes are sequenced, and as annotation of existing genomes improves (e.g., [139–141]). Data sets relevant to the reconstruction of LECA will amass quickly, for example, as a product of the Earth Biogenome Project [142] and the associated Darwin Tree of Life [143] and Aquatic Symbiosis Genomics [144] projects, and from metagenomic sampling of microbial diversity (e.g., [9,145–147]). Furthermore, as models of sequence evolution continue to improve [23,128–131,148,149] phylogenetic and phylogenomic relationships will be re-evaluated. Improved homology detection methods, particularly structure-based methods utilising the latest AI approaches [150], will resolve homology relationships, trigger re-analysis of the relative contributions of different prokaryotes to LECA, and further improve comparative phylogenetic analyses [151]. Such approaches will also help to clarify patterns of homology between divergent eukaryotic genes, leading to a reassessment of when and how ortholog groups were acquired within the eukaryotic radiation. For these reasons, attempts to define a LECA gene repertoire are a “hostage to fortune”; as new data become available and methods improve; revision and tools to enable revision are needed. We provide a set of recommendations that could serve as a pathway forward and sketch an analytical approach allowing reconciliation of different LECA data sets (**Fig 3A and 3B**).

We are advocating for a large-scale, cooperative, and community-minded approach to inferring a full LECA gene set (**Box 2**). This reconstruction requires the accurate estimation of eukaryotic orthologous gene family relationships [152], followed by the identification of sister group relationships in order to identify and polarise gene duplications and, when appropriate, infer prokaryotic ancestry (**Fig 3A**). Fast approximations of ortholog clustering are possible using automated methods [153–155], but these approaches are error-prone—they can classify paralog-containing clusters as orthologs (under-splitting), separate in-paralogs/recent duplications from their bona fide orthologs (over-splitting) [156], and erroneously split orthologous groups due to high levels of sequence divergence (also over-splitting) [157]. As a consequence, some researchers combine fast ortholog clustering with manual curation [158,159], a practice that can mitigate such issues but also introduces subjectivity. The greater part of these curation process (and the subjectivity involved) is lost to the wider scientific record and can produce data sets that are difficult to analyse, compare, and critically assess [160]. Providing access to the data from these “chains” of analyses will be important, especially for the systematic integration of new data sets which allows for the revision of ortholog classifications (see **Box 2** recommendations and **Fig 3B**).

**Box 2: Aspirational standards for a community approach to LECA analyses**

**For both large-scale and system-specific reconstructions**

- Eukaryote-wide phylogenomic analyses should make phylogenetic trees, amino acid sequence alignments, and HMMs representing gene clusters, along with the underlying methods, easily accessible (e.g., through data repository services).
- Trees, alignments, and HMMs representing gene clusters should be accessible for cross comparison, i.e., presented in tractable file formats (e.g., NEWICK, FASTA, HMMs, respectively).
- Source and assembly versions of the genome data sets used for analyses should be indicated, ideally with date of access or annotation version available.
- Sequence data decontamination processes should be described, and the resulting genome/proteome made available.
- LECA repertoire estimations should account for ancient gene duplications both in the prokaryotes (pre-LECA) and within the eukaryotes, thus separating gene families into eukaryotic ortholog clusters where possible, such that paralog relationships are identifiable.
- For each LECA gene repertoire reconstruction, eukaryotic phylogenies and root hypotheses should be clearly stated and, optimally, various alternatives should be considered so that different ancestral complements can be compared.
- If ancestral gene repertoire reconstructions are estimated, alternative approaches should be compared (e.g., Dollo parsimony, maximum likelihood [with different birth/death models], Bayesian, and reconciliation approaches).
- Different methods of ancestral gene repertoire reconstruction will provide variant estimates of eukaryote-to-eukaryote HGT. This factor should be acknowledged and targeted phylogenetic analysis to validate candidate HGT families is advised.
- Automated ortholog assessment methods should be supervised and/or validated. Correction and validation processes should be recorded in a data accessible manner (e.g., differences between processed ortholog sets should be made available).
- The process of ortholog amendments should be described.

**Specific to large-scale, all-systems reconstruction of LECA**

- LECA repertoire estimations should identify gene sets where there is no phylogenetic resolution or there are too few alignable sites to allow conclusive phylogenetic analyses.
- For each LECA gene repertoire reconstruction, the proportion of LECA gene families for which a prokaryotic donor can or cannot be pinpointed should be indicated.
- The approach used to account for eukaryotic paralog expansions, i.e., whether expanded eukaryotic families are counted as a single entity or individually by duplicate number in LECA should be clearly stated when assigning relative percent prokaryotic contributions to LECA.
- For hypotheses invoking multiple prokaryotic donors into LECA, the relative proportion of phylogenies which support each purported prokaryotic donor group should be indicated.
- Having established the set of prokaryotic donors to LECA, ancestral gene repertoires should be used to systematically test for the role of HGT pre-LECA.

Once ortholog groups are established, it is in principle possible to compare these groups with homologous gene clusters from prokaryotes and then map the origin of eukaryotic gene families onto the prokaryotic tree of life (**Fig 3C**). Such analyses are complicated by ever-growing data sets that often result in sequence alignment sizes that restrict the use of sophisticated phylogenetic methods, in turn necessitating phylogenetically informed down-sampling. Nonetheless, ancestral state reconstruction using parsimony, Bayesian, or maximum likelihood methods [161] can be used to map gene family acquisition to a species tree, each giving somewhat different views of how gene content is inherited across the tree [162]. Some methods also allow for joint species tree/gene tree reconciliation analyses using likelihood-based inference, enabling mapping of gene repertoires onto species phylogenies [163]. Understanding the pattern of gene flow identified by these differing approaches requires further investigation of individual gene phylogenies to identify eukaryote-to-eukaryote or prokaryote-to-eukaryote HGT, as well as sequence data contamination, to avoid overestimating gene presence in ancestors such as LECA. The inference of sister group relationships informed by ancestral state reconstruction between prokaryotic gene clusters and eukaryotic orthologs can allow understanding of when and how gene families were acquired by eukaryotes (e.g., HGT, endosymbiosis, de novo gene acquisition, and gene duplication). Ancestral gene complements can also be reconstructed under various eukaryotic phylogenies and root hypotheses so that different ortholog gene family repertoires can be compared, which is important when there is uncertainty regarding topological relationships within the species phylogeny (e.g., [164–167]) (**Fig 3C**).

Ortholog detection is a challenge that increases in complexity with gene family size, gene loss events, and data asymmetry, e.g., the comparison of highly sampled taxonomic groups with groups with few genome sequences. The goal of the community-based “Quest for Orthologs” initiative [168] is to evaluate the strengths and weaknesses of tools for identifying orthologous gene families; members are committed to open exchange of methods and approaches supported by shared benchmarking tools enabling cross validation [169]. This is exactly the approach needed for the study of LECA, a community that provides tools and benchmarks, and sets standards for data sharing.

An aspect of this community approach would be a clear framework for systematic comparison of different LECA reconstructions. For example, given a set of putative eukaryote-wide orthologs, it is possible to generate individual hidden Markov models (HMMs [170–173]) that can be used to define each individual ortholog cluster. Current HMM methods can also be tailored to exclude certain sequences thereby allowing analyses to be targeted for specific orthologs while excluding paralogs and xenologs [174]. Refined HMM sets can then be used to compare [172] and add additional genomes to the comparative data set, allowing for iterative revision of both the ortholog groups and the HMMs themselves (**Fig 3B**).

One advantage of HMM-to-HMM comparison methods (e.g., [172]) is that they make it possible to compare and, if needed, reconcile different LECA ortholog data sets. Such an approach can be used to systematically revise ortholog gene families as new LECA data sets are released. But the data must be accessible to allow systematic comparisons (**Box 2** and **Fig 3B**). Ideally, such an endeavour would be mounted as a web-based database for the community, allowing updates and corrections. Ortholog classifications can then be improved iteratively with more data and increasing engagement (**Fig 3B**). The history of source data and the revision chain would therefore be available for each gene family so researchers could view how ortholog assignments have progressed. In addition, the orthogroups identified as part of this community effort could be of wider use. Validated orthogroups could for example be used as an update to the KOG database that provides the core of the eukaryotic level orthology in EggNOG [175], thereby providing feedback to the larger comparative genomics community and making orthogroup classifications readily usable for gene annotation and other informatic applications.

A related consideration is that the data sets arising from LECA-scale analyses are contained within the supplementary materials of complex publications. Here, the traditional publication model fails the phylogenomic endeavour because these data are not easily accessible or standardised for systematic comparison. Such comparisons are fundamental for understanding how to improve estimation of LECA gene repertoire sets. Given the data problems outlined, as a community, we must strive for data release, accessibility and analysis standards that allow for systematic comparison.

We have provided recommendations (**Box 2**) and sketched a pathway (**Fig 3A and 3B**) to enable an accessible large-scale, all-taxa reconstruction of LECA, providing access to cross-comparison and facilitating iterative improvement. To enable this endeavour, we also advocate for the development of web-based database resources to support such interactions (e.g., [176]). As genome sampling increases and ortholog sets are corrected, LECA gene complement estimation could be iteratively revised (e.g., LECA 2.0, etc.; ideally with a release schedule outlined so researchers in the field can plan accordingly). Now is the time for the community to start building LECA-specific tools and resources for handing the complicated task of data analysis required to resolve the gene repertoire of LECA in a way that caters to differential approaches and perspectives while also making iterative chains of phylogenomic analyses available. We recognise that many groups will continue with focused analyses of individual cellular systems. These analyses will complement, and can integrate with, any large-scale LECA reconstruction, providing important ground-truthing data sets for the annotation and manual correction phases outlined in **Fig 3B**. Furthermore, large-scale LECA reconstruction will identify groups of genes that are especially difficult to resolve using bioinformatic pathway-based approaches which therefore need focused analyses, making these approaches both complementary and iterative. Many of the recommendations regarding data sharing, standards, and transparency (**Box 2**) apply equally to both types of effort.

### Beyond eukaryogenesis—The wider value of reconstructing LECA

The origin of the eukaryotic cell laid the foundation for a vast diversification of biological forms leading to additional major evolutionary transitions. As a resolved LECA gene repertoire provides a baseline from which to infer lineage-specific evolutionary changes within the eukaryotes, this data set will allow researchers to address a multitude of questions, both evolutionary and cell biological in nature.

A LECA gene set will allow study of the evolutionary dynamics during the early diversification of the major eukaryotic groups, including the contributions of gene gain, loss, duplication, HGT, and domain rearrangement (i.e., gene-fusions and -fissions). Such data will also support a range of downstream analyses, for example, providing expected ortholog distribution maps for evaluating eukaryotic genome assembly completion, similar to the approaches applied in BUSCO [177] and OMArk [178]. Resolved ortholog relationships will also be an important resource for concatenated multi-gene phylogenomic analysis (e.g., [179]) underpinning further investigations of the eukaryotic tree. Finally, a LECA repertoire provides a starting gene repertoire from which to infer the evolution of nearly all extant eukaryotic cellular functions. This includes the origin and spread of photosynthetic organelles [180,181], the repeated evolution of pathogenicity (e.g., [182–185]), and the multiple origins of multicellular forms such as plants, animals, fungi, and seaweeds [186].

The LECA gene set should also serve as baseline data for fundamental cell biological inquiries aiming to move beyond standard model organisms (e.g., yeast, animal, or plant). Such organisms are unrepresentative of the diversity of eukaryotic traits and cellular forms, although comparison of the 3 groups is, of course, important. The genes, proteins, and processes found in LECA can be considered ancient and are potentially generalizable as features of “the eukaryotic cell.” Furthermore, the LECA analyses proposed here would identify conserved gene families present across the eukaryotes for which there is no known functional annotation. Many of these may turn out to be jötnarlogs—genes with patchy distributions, absent in model organisms, but present in diverse organisms of medical or ecological importance (e.g., [71,187]). Such data are important, for example, when researchers wish to identify a gene present in a group of pathogens/parasites with no host-encoded homologous protein as a putative drug target. Finally, a LECA gene repertoire facilitates investigation of co-occurrence patterns between uncharacterised core systems and known cell functions (e.g., [102,188]), thereby providing clues regarding function. The results of a wide range of LECA analyses can be compared to large-scale knockout libraries in model systems providing further information on function and evolution [189].

### Conclusion

Resolving the early evolution of the eukaryotic cell remains a huge challenge [21]. Given its importance and antiquity, we have more hypotheses than definitive data. Consequently, every detail upon which a consensus is reached can push inferences towards one eukaryogenesis scenario over another, or help us to resolve a key factor in the early evolution of eukaryotes. An estimation of the LECA gene repertoire is a foundational data set for testing pivotal ideas about how the early eukaryotic cell evolved, providing an end state at which all eukaryogenesis models need to arrive and a starting point for understanding the evolution of major eukaryotic groups and their cellular systems. A community-wide effort to define LECA in terms of cell biology and gene repertoire will permit informed comparisons of different models so that they can be judged on their relative merits. This is a complex task, one in which different approaches and new data can radically alter patterns. Such investigation can therefore only realistically move forward through systematic community engagement with adherence to shared standards. To that end, we have outlined recommendations for data analyses and accessibility to allow for systematic comparisons. We have also sketched out an analytical pathway that would allow for the cross comparison of LECA data sets given the changing availability of data (**Fig 3**). Our hope is that this framework will be useful for individual research teams and discipline-wide consortia alike, and that the ideas presented herein about how these data should and could be used will trigger new ways of thinking about the problem of eukaryogenesis and early eukaryotic cell evolution (**Box 2**).

## Abbreviations

ESP: eukaryote signature protein
FECA: First Eukaryotic Common Ancestor
HGT: horizontal gene transfer
HMM: hidden Markov model
LECA: last eukaryotic common ancestor
